# Supplementary material for: Impaired Bile Acid Metabolism and Gut Dysbiosis in Mice Lacking Lysosomal Acid Lipase
Source: Cells. 2021 Oct 1;10(10):2619. doi: 10.3390/cells10102619 (PMC8533808; doi:10.3390/cells10102619)
Supplement: Supplementary file 1 [file cells-10-02619-s001.zip › cells-1363181-Suppl.pdf]

Supplementary Table S1: Primer sequences used for quantitative real time PCR analysis

**Gene name**	**Primer sequences (5' to 3')**
Fibroblast growth factor 15 (*Fgf15*)	F- GCC ATC AAG GAC GTC AGC A<br>R- CTT CCT CCG AGT AGC GAA TCA
Cholesterol 7 alpha-hydroxylase (*Cyp7a1*)	F- GGG CAT CTC AAG CAA ACA CCA<br>R- CGG GAC TGA TCT AGA GGG GGA
Fibroblast growth factor receptor 4 (*Fgfr4*)	F- TTG ATG TCT GTT GTC TTC AGG<br>R- CCC ACA TAC AGT GGC TGA AAC
Sterol 12-alpha-hydroxylase (*Cyp8b1*)	F- AAG GCT GGC TTC CTG AGC TT<br>R- AAC AGC TCA TCG GCC TCA TC
Bile salt export pump (*Bsep/Abcb11*)	F- CTG CCA AGG ATG CTA ATG CA<br>R- CGA TGG CTA CCC TTT GCT TCT
Sodium/taurocholate co-transporter 3 (*Ntcp/Slc10a1*)	F- ATG ACC ACC TGC TCC AGC TT<br>R- GCC TTT GTA GGG CAC CTT GT
Cytochrome P450, family 2, subfamily c, polypeptide 70 (*Cyp2c70*)	F- TGG CTT TCT CAG CAG GAA GAA<br>R- AAC TGG CTT GGT GTC GAT GT
Cytochrome P450, family 27, subfamily a, polypeptide 1 (*Cyp27a1*)	F- GCC TTG CAC AAG GAA GTG ACT<br>R- CGC AGG GTC TCC TTA ATC ACA
Beta-klotho (*β-Klotho*)	F- ACG GCT GGT TCA CAG ATA GCT<br>R- CAT CAA ATT TTA TTG CTT GAA
Oxysterol 7-alpha-hydroxylase (*Cyp7b1*)	F- CTG CAG TCA ACA GGT CAA A<br>R- GCC TCA GAA CCT CAA GAA TAG
Apical sodium-dependent bile acid transporter (*Asbt/Slc10a2*) (Ileal)	F- GTA CAA TGG TGG AGC ACA GC<br>R- GTG CCT GGA TCA TTG AAC CC
Ileal fatty acid binding protein 6 (*I-Babp/Fabp6*) (Ileal)	F- GGC AAA GAA TGT GAA ATG CAG<br>R- CCG AAG TCT GGT GAT AGT TGG
Organic solute transporter a/b (*Osta/b*) (Ileal)	F- AGT TTG CTC TGT TCC AGG TGC<br>R- TGT TAG CCA AGA TGG AGA AAA

Supplemental Figure S1

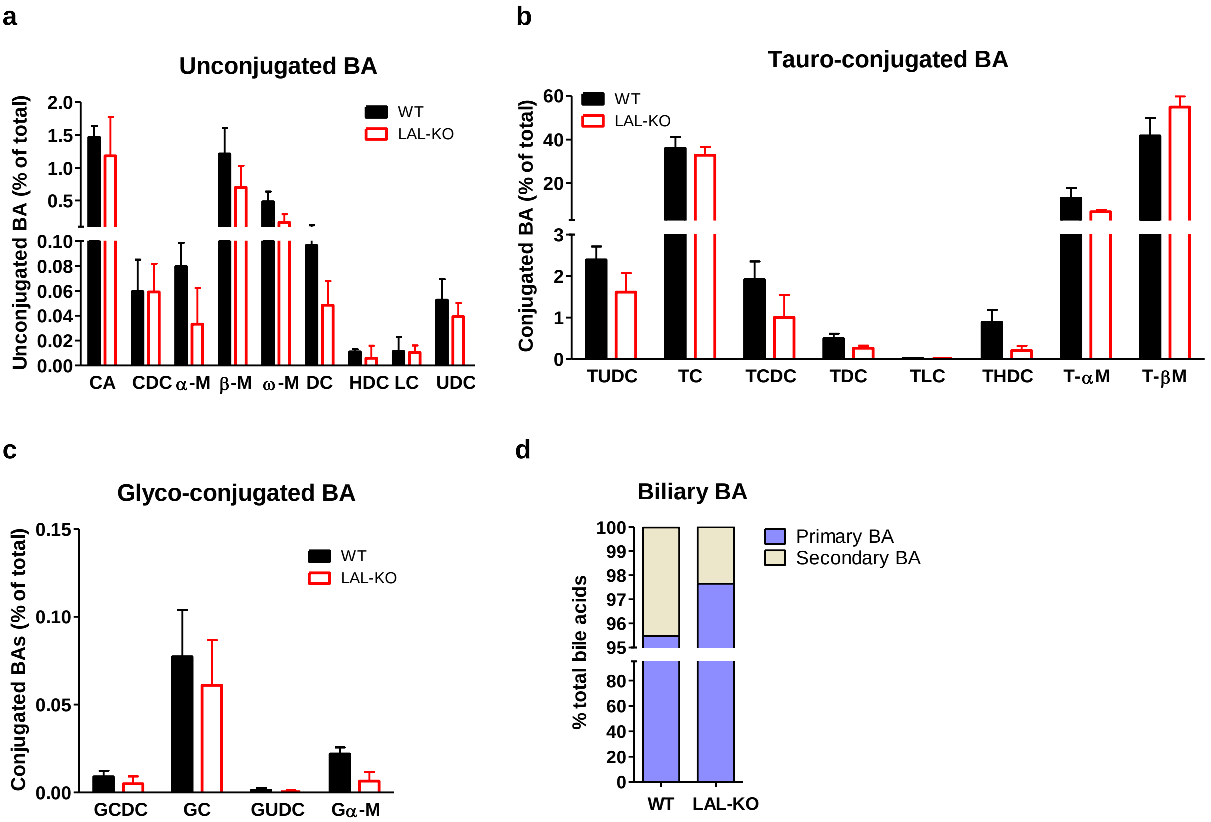

Figure S1: Biliary bile acid composition in WTD-fed LAL-KO mice: Biliary (**a**) unconjugated, (**b**) tauro-conjugated, and (c) glyco-conjugated bile acids. (**d**) Proportion of primary and secondary biliary bile acids of WTD-fed male mice (12-14 weeks of age). Primary bile acids include free and conjugated forms of CA, CDCA, ά-MCA, and β-MCA, whereas secondary bile acids include DCA, LCA, ω-MCA, and UDCA.
